# Additional Editorial Board members

**DOI:** 10.4314/gmj.v55i1.1

**Published:** 2021-03

**Authors:** David Ofori-Adjei

The Ghana Medical Journal extends a special welcome to new members appointed to the Editorial Board. The members are to complement the work of the current membership of the Board.

**Dr John Amuasi**

Senior Lecturer, Department of Global Health, School of Public Health and Head, Department of Community Health, School of Medicine and Dentistry, Kwame Nkrumah University of Science and Technology, Kumasi, Ghana Leader, Global Health and Infectious Diseases Research Group, Kumasi Collaborative Center for Research in Tropical Medicine (KCCR at KNUST) and Bernhard Nocht Institute of Tropical Medicine, Hamburg, Germany.

**Professor Joe-Nat Clegg-Lamptey**

Department of Surgery, University of Ghana Medical School, College of Health Sciences, Accra, Ghana

**Professor Adam Gyedu**

Department of Surgery, School of Medical Sciences, Kwame Nkrumah University of Science & Technology, Kumasi, Ghana and the University Hospital, Kumasi, Ghana.

**Dr Samuel Blay Nguah**

Senior Lecturer, Department of Child Health, School of Medical Sciences, Kwame Nkrumah University of Science and Technology, Kumasi, Ghana and Head of Cardio-Pulmonary Unit, Komfo Anokye Teaching Hospital, Kumasi, Ghana

**Nana Ayeguah Hagan Seneadza**

Lecturer, Department of Community Health, School of Public Health, College of Health Sciences, University of Ghana, Accra, Ghana

**Professor Alfred Yawson**

Department of Community Health, School of Public Health, College of Health Sciences, University of Ghana and Department of Biostatistics, School of Public Health, College of Health Sciences, University of Ghana, Accra, Ghana.

